# Investigation of Adhesion Performance of Wax Based Warm Mix Asphalt with Molecular Dynamics Simulation

**DOI:** 10.3390/ma15175930

**Published:** 2022-08-27

**Authors:** Chao Peng, Hanneng Yang, Zhanping You, Hongchao Ma, Fang Xu, Lingyun You, Aboelkasim Diab, Li Lu, Yudong Hu, Yafeng Liu, Jing Dai, Zhibo Li

**Affiliations:** 1 Faculty of Engineering, China University of Geosciences, Wuhan 430074, China; 2 Department of Civil and Environmental Engineering, Michigan Technological University, Houghton, MI 49931-1295, USA; 3 School of Civil and Hydraulic Engineering, Huazhong University of Science and Technology, Wuhan 430074, China; 4 Department of Civil Engineering, Aswan University, Aswan 81542, Egypt; 5 Key Laboratory of Advanced Technology for Specially Functional Materials, School of Materials Science and Engineering, Wuhan University of Technology, Wuhan 430070, China

**Keywords:** molecular dynamics, wax warm mix asphalt, contact angle test, pull-off test, Fourier transform infrared spectroscopy, adhesion work

## Abstract

Compared with traditional hot mix asphalt (HMA), wax based warm mix asphalt (WWMA) can be mixed with the aggregate at a lower temperature and achieve the desired compaction. However, the adhesion performance of WWMA on aggregate is uncertain. To evaluate the adhesion performance of asphalt and aggregate, researchers used contact angle test, pull-off test, and ultrasonic washing experiments. However, these tests cannot adequately explain the microscopic mechanism of the interface between asphalt and aggregate. Molecular dynamics (MD) can better explain the adhesion mechanism of asphalt aggregates because they can be simulated at the molecular scale. So, the purpose of this research is to use the MD method to study the adhesion performance between WWMA and aggregate. Two aggregate oxides (CaCO_3_ and SiO_2_) models, the matrix asphalt model and WWMA models, were built in Materials Studio (MS) software. The adhesion work of asphalt and aggregate oxides was calculated. With the increase of wax modifier content, the adhesion work of asphalt and aggregate oxides (CaCO_3_ and SiO_2_) first increases and then decreases. When the wax modifier is increased to 3 wt%, the adhesion works of the WWMA-SiO_2_ and WWMA-CaCO_3_ increase by 31.2% and 14.0%, compared with that of matrix asphalt. In this study, the accuracy of the MD calculation result was verified by the pull-off experiments and the contact angle experiments. WWMA was prepared by a high-shear mixer emulsifier. In the pull-off experiments and the contact angle experiments, the tensile strength and the adhesion work between the aggregate and the asphalt containing 3% wax modifier reaches peak values. These values are 140.7% and 124.9%, compared with those between the aggregate and the matrix asphalt. In addition, the results of the pull-off experiments and the contact angle experiments are in good agreement with that of the MD simulation. Finally, Fourier transform infrared spectroscopy (FTIR) shows that the carbonyl content of WWMA is greater than that of matrix asphalt. It explains well that the wax modifier promotes the adhesion between asphalt and aggregate. This paper provides an important theoretical basis to understand the adhesion performance of WWMA and aggregate.

## 1. Introduction

Compared with hot mix asphalt (HMA), warm mix asphalt (WMA) can be mixed with aggregate at lower temperatures and reduces energy consumption. The mixing temperature of WMA is usually 20 to 40 °C lower than that of HMA [1,2,3,4]. WMA can improve the workability of asphalt binders and reduce the emission of harmful gases. It is a green environmental protection technology with low energy consumption and emission.

There are three main methods for producing WMA by use of synthetic or organic additives, which are zeolite [5,6], surfactant [7,8] and wax [9,10]. The zeolite and wax reduce the mixing temperature of asphalt and aggregate by reducing the viscosity of the asphalt. The surfactant reduces the mixing temperature of asphalt aggregate by reducing the surface tension between asphalt and aggregate. Compared with surfactant and zeolite, wax can improve the moisture susceptibility and anti-rutting performance of asphalt mixtures [11,12]. Therefore, wax modifiers are more widely used to reduce the mixing temperature of asphalt and aggregate.

The adhesion performance of asphalt and aggregate is directly related to the water damage resistance of asphalt pavement [13]. Enhancing the adhesion of wax based warm mix asphalt (WWMA) and aggregate is of great significance for prolonging the service life of WWMA pavement. In recent years, many researchers have studied the adhesion performance of WWMA and aggregate. Akhtar et al. [14] evaluate the adhesion performance of Sasobit (a type of wax modifier) modified asphalt and aggregate by atomic force microscope (AFM). They found that Sasobit improves the adhesion between asphalt and aggregate. Wen [15] calculated the adhesion work of Sasobit modified asphalt and matrix asphalt on aggregate using surface free energy to evaluate the adhesion strength. The result shows that the adhesion strength between Sasobit modified asphalt and aggregate is better than that of matrix asphalt and aggregate. Yang et al. [16] measured the contact angle of different liquids on the surfaces of penetration grade 60–70 asphalt (Pen 60–70 asphalt) and WWMA to calculate the surface energy and adhesion work of asphalt and aggregate. They found that the adhesion work of WWMA on aggregate is increased by 6% compared with Pen 60–70 asphalt on aggregate. This shows that WWMA has better adhesion strength than Pen 60–70 asphalt. To sum up, most of the above researchers used macroscopic experiments to study the adhesion performance between WWMA and aggregate. However, the microscopic mechanism related to adhesion performance between WWMA and aggregate is still unclear.

In recent years, MD simulation has been an effective method to study the microscopic mechanism of material components from the perspective of molecular motion. Many researchers have evaluated the adhesion performance between asphalt and aggregate by calculating the adhesion work of the asphalt-aggregate model in MD simulation. Tarefder et al. [17] used the MD method to determine the thermodynamic properties of asphaltene before and after oxidative aging. The results showed that the glass transition temperature is related to the viscosity, hardness, and rigidity of asphalt, and decreases with the increased oxidative aging degree. Qu et al. [18] studied paraffin’s effect on the mechanical properties of asphalt binder through MD simulation. The results showed that paraffin could reduce the high-temperature stability and self-healing rate of asphalt binder. Chu et al. [19] established asphalt-aggregates (quartz and calcite) models in MS software. They discovered that the adhesion strength of asphalt and calcite is greater than that of asphalt and quartz. Gao et al. [20] constructed the interface models of asphalt and four aggregates (quartz, calcite, albite, and microcline) in MS software. They revealed that the adhesion work of asphalt and microcline is larger than asphalt and other aggregates. Xu et al. [21] adopted the ReaxFF force field to simulate the interaction between the main molecules in asphalt and aggregate (SiO_2_). They found that the adhesion work of phenol molecules and SiO_2_ is much larger than that of other molecules and SiO_2_. The above researches show that MD has been widely used to study the adhesion performance of asphalt-aggregate. However, there are few studies on the adhesion performance of WWMA and aggregate by using the MD method.

In this study, we studied the adhesion between WWMA and aggregates using the MD method. Twelve different molecules were first selected to represent the asphalt component models. Then, wax molecules were chosen as the wax modifier to construct WWMA models. The adhesion work between asphalt and two aggregate oxides (CaCO_3_ and SiO_2_) was calculated by MD simulation. Then, the accuracy of the MD simulation calculation results was verified by the pull-off experiments and the contact angle experiments. The Fourier transform infrared spectroscopy (FTIR) was conducted to reveal the chemical mechanism of adhesion performance between WWMA and aggregate.

## 2. Experiments and Methods

### 2.1. MD Simulation Models

#### 2.1.1. Force Field Selection

MD simulation is to analyze the basic properties of materials by simulating the motion and interaction of material atoms. The principles of MD are based on statistical mechanics and Newton’s laws of motion. Condensed-phase Optimized Molecular Potential for Atomistic Simulation Studies (COMPASS) can accurately simulate the interaction of organic and inorganic molecules. The COMPASS force field has been successfully applied to simulate the adhesion performance of asphalt and aggregate in previous study [22]. The COMPASS force field was selected in this work and can be described by Equations (1)–(3) [23].

(1) $$E_{total}=E_{val}+E_{non-bond}$$

(2) $$E_{val}=E_{b}+E_{\theta }+E_{\varphi }+E_{\chi }+E_{bb^{\prime }}+E_{b\theta }+E_{b\varphi }+E_{\theta \varphi }+E_{\theta \theta^{\prime }}+E_{\theta \theta^{\prime }\varphi }$$

(3) $$E_{non-bond}=E_{elec}+E_{LJ}$$

where *E_total_* is total potential energy, *E_val_* is valence state energy, *E_non-bond_* is non-bond energy, *E_b_* is bond stretching energy, *E_θ_* is angular bending energy, *E_φ_* is internal torsion energy, *E_χ_* is out-of-plane bending energy, *E_bb′_*, *E_bθ_*, *E_bφ_*, *E_θφ_*, *E_θθ′_*, and *E_θθ′φ_* are the interactions of crosscoupling terms, *E_elec_* is Coulomb electrostatic energy, and *E_LJ_* is the van der Waals energy.

#### 2.1.2. Matrix Asphalt Model

**Figure 1:**
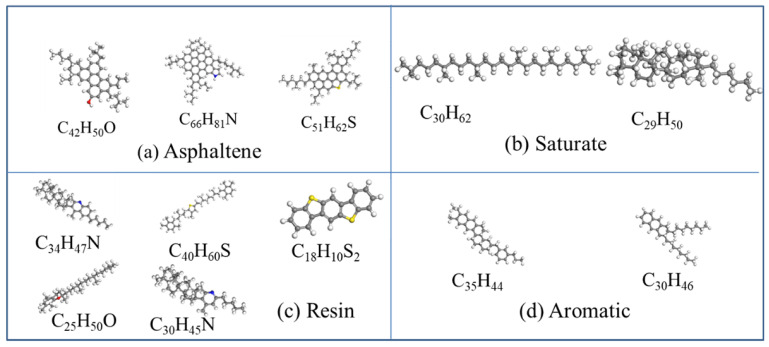
(**a**) Asphaltene molecular models; (**b**) Saturate molecular models; (**c**) Resin molecular models; (**d**) Aromatic molecular models.

The American Society for Testing and Materials (ASTM, West Conshohocken, PA, USA) D4124-09 proposed the SARA (saturated, aromatic, resin, and asphaltene) classification scheme. Li and Greenfield [24] developed three 12-component asphalt models, namely AAA-1, AAK-1 and AAM-1. Compared with other model systems, the density and thermal expansion coefficient of the AAA-1 model system are closer to the experimental data of Jones et al. [25]. In this paper, the AAA-1 asphalt model is selected as the model of matrix asphalt. The specific parameters of matrix asphalt are listed in Table 1. The constructed four-component molecular models of matrix asphalt are shown in Figure 1.

**Figure 2:**
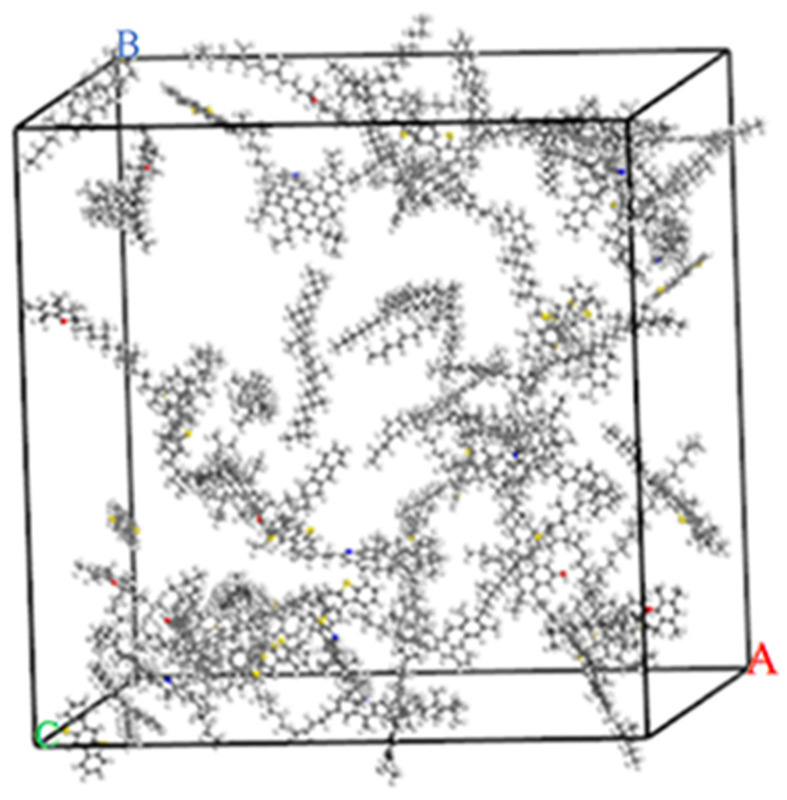
Matrix asphalt model.

First, the Amorphous Cell Calculation module in the MS software was used to construct an amorphous asphalt model. The initial density of asphalt was set to 0.1 g/cm^3^. Then, the asphalt was placed in a periodic cube box. The constructed matrix asphalt model is shown in Figure 2.

#### 2.1.3. WWMA Models

**Figure 3:**
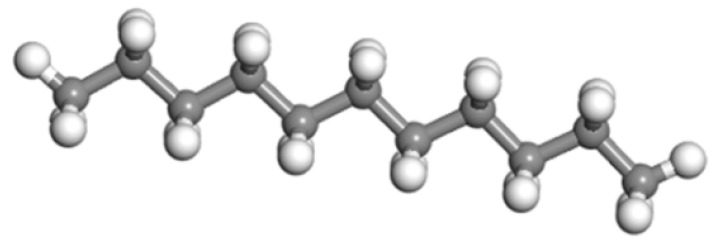
Wax molecular model.

The wax molecular model constructed by Samieadel et al. [26] was used in this study. The molecular formula of wax is C_11_H_24_. The molecular model of wax is shown in Figure 3.

**Figure 4:**
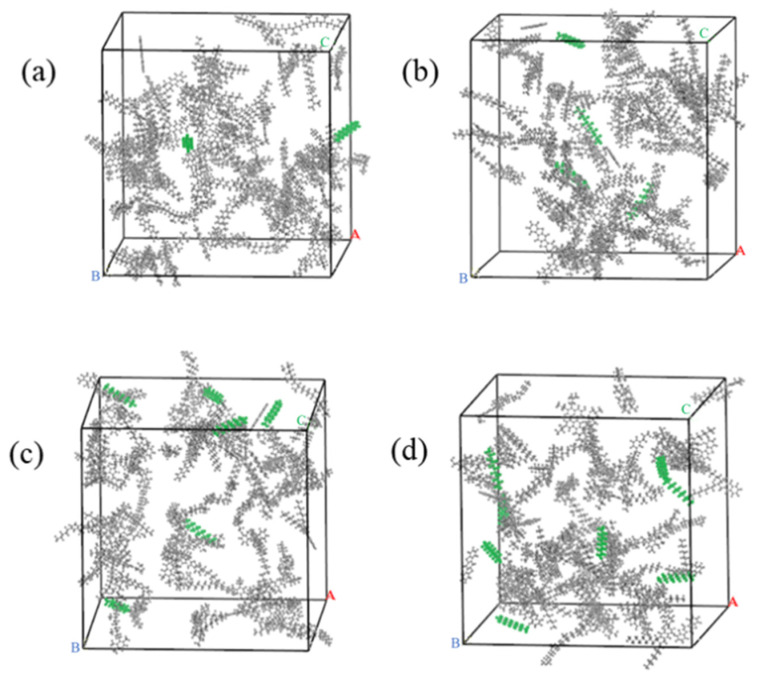
(**a**) WWMA-1 model; (**b**) WWMA-2 model; (**c**) WWMA-3 model; (**d**) WWMA-4 model.

The molar mass of the matrix asphalt model is 32,710.4 g/mol. The molar mass of wax is 156.4 g/mol. A total of 2, 4, 6, and 8, mol of wax molecules were added to the matrix asphalt to construct WWMA models. The WWMA models are shown in Figure 4.

#### 2.1.4. Model Optimization

**Figure 5:**
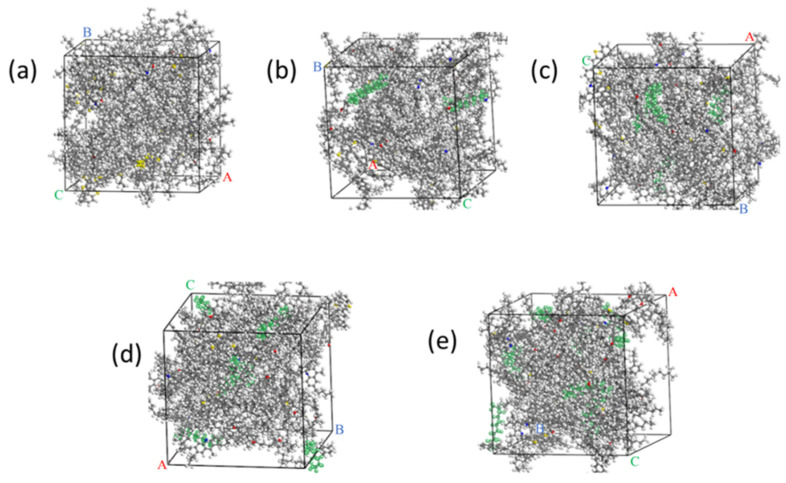
(**a**) Optimized matrix asphalt model; (**b**) Optimized WWMA-1 model; (**c**) Optimized WWMA-2 model; (**d**) Optimized WWMA-3 model; (**e**) Optimized WWMA-4 model.

The matrix asphalt and WWMA models were geometrically optimized in the Forcite module. In order to minimize the energy of the asphalt system, the asphalt models were subjected to 5000 iterations under the COMPASS force field to achieve geometric optimization. Then, the asphalt models were used for dynamics simulation in the NPT (constant pressure and constant temperature) ensemble and the NVT (constant temperature and constant volume) ensemble for 100 ps to be stable. Finally, asphalt models with stable volume and energy fluctuations were obtained. The asphalt models after geometric optimization and dynamics simulation are shown in Figure 5. Compared with Figure 2 and Figure 4, the structure of each asphalt model is more compact after geometric optimization and dynamics simulation.

### 2.2. Adhesion Work

#### 2.2.1. Single Cell Oxide Model

**Figure 6:**
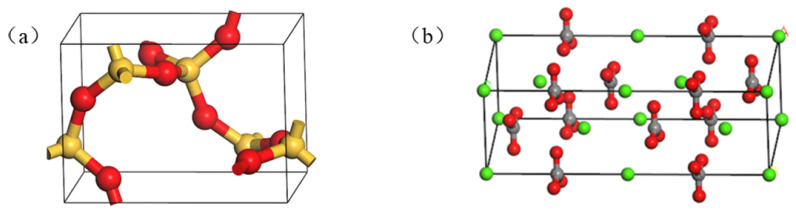
(**a**) The single-cell model of SiO_2_; (**b**) The single-cell model of CaCO_3_.

Limestone, basalt, granite, diabase, and amphibolite, are mostly used as aggregates. The aggregates mainly contain SiO_2_, CaCO_3_, Al2O_3_, MgO, and other components. In this paper, two oxide molecules (SiO_2_ and CaCO_3_) were selected in MD simulation. The SiO_2_ and CaCO_3_ models are shown in Figure 6. The single-cell model parameters of SiO_2_ and CaCO_3_ are listed in Table 2.

#### 2.2.2. Oxide Supercell Model

**Figure 7:**
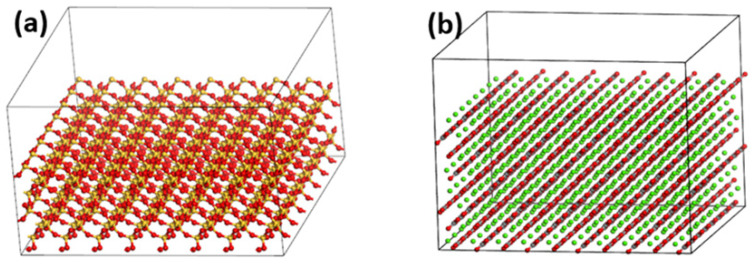
(**a**) The supercell model of SiO_2_; (**b**) The supercell model of CaCO_3_.

In MS, the aggregate model is represented by a supercell. First, the single-cell oxide was cut along the (0,0,1) direction to expose the surface. Then, the Supercell function in the Build toolbar was used to build the supercell model. A 10 Å vacuum layer was inserted in the Z direction of the constructed supercell model. Finally, geometric optimization was used to optimize its structure. The SiO_2_ supercell model and the CaCO_3_ supercell model are shown in Figure 7.

#### 2.2.3. Calculation of Adhesion Work

The asphalt-aggregate interface system was constructed by attaching an asphalt layer to the surface of the aggregate. Then, a 30 Å vacuum layer was placed on top of the asphalt layer. In the MD simulation, the asphalt-aggregate models were first geometrically optimized. Then, the dynamic balance of the NVT ensemble of 100 ps was performed to optimize the structure of the asphalt-aggregate models further. In this study, all the MD simulated temperatures were performed at 298 K, and the specific parameters refer to the literature [27].

In order to quantitatively evaluate the adhesion strength of asphalt and aggregate, this study calculated the adhesion work of the asphalt-aggregate model. Adhesion work is defined as the energy required to separate an interface per unit area into two free surfaces in a vacuum [28]. The adhesion work corresponds to the adhesion strength of the asphalt-aggregate system. The adhesion work of asphalt-aggregate per unit area (*W_MB_*) and the adhesion work of the asphalt-aggregate (∇*E_MB_*) can be calculated by Equations (4) and (5) [29].

(4) $$W_{MB}=\frac{\nabla E_{MB}}{A}$$

(5) $$\nabla E_{MB}=E_{M}+E_{B}-E_{MB}$$

In Equation (4), *A* is the contact area between asphalt and aggregate. In Equation (5), *E_M_* and *E_B_* are the potential energy of aggregate and asphalt at thermodynamic equilibrium, respectively. *E_MB_* is the asphalt-aggregate system in thermodynamics potential energy at equilibrium. Since the *A* of the asphalt-aggregate system is constant, ∇*E_MB_* can directly reflect the adhesion work of the asphalt-aggregate system.

### 2.3. Macroscopic Adhesion Performance Experiment

#### 2.3.1. Raw Materials

In this study, the used 90# matrix asphalt is produced by Maoming Petrochemical Company in Guangdong Province, China. Its physical properties are listed in Table 3. The wax modifier used to prepare WWMA comes from AkzoNobel Co., Ltd., Jiaxing, China. Wax modifier is light white solid particles. The physical properties of the wax modifier are listed in Table 4.

#### 2.3.2. Preparation of WWMA

First, 90# matrix asphalt was heated. Then, 1 wt%, 2 wt%, 3 wt%, and 4 wt%, of wax modifier were mixed with the hot 90# matrix asphalt, respectively. The WWMA were prepared by a high-shear mixer and stirred at a speed of 5000 r/min for 15 min. The WWMA samples containing 1 wt%, 2 wt%, 3 wt%, and 4 wt%, of wax modifier are referred to as WWMA-1, WWMA-2, WWMA-3, and WWMA-4, respectively.

#### 2.3.3. Pull-Off Test

**Figure 8:**
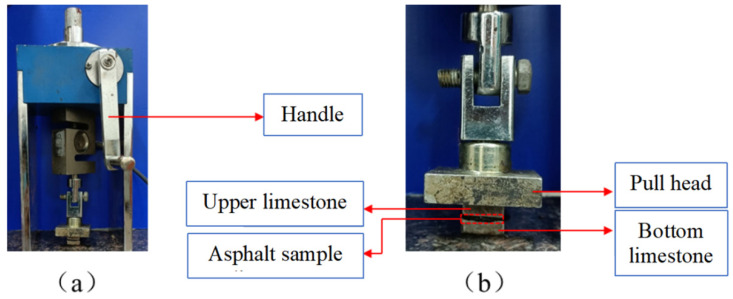
(**a**) The photo of the pull-off instrument; (**b**) The photo of asphalt sample and the pull head.

In this experiment, the ZQS6 pull-off instrument was used to measure the tensile strength. The pull-off instrument is shown in Figure 8. First, limestone was cut into 1.5 cm × 1.5 cm × 1 cm cubes (the main component is CaCO_3_). Then, the limestone cubes and asphalt were placed in an oven at 170 °C. When the asphalt was heated into a fluid state, asphalt was dropped on to the surface of the bottom limestone cube. After the asphalt spreads evenly to the surface of the bottom limestone, the upper limestone was placed on the bottom limestone. When the asphalt sample was cooled to 20 °C, the upper and bottom limestone cubes adhered to the pull head and the marble slab with epoxy glue, respectively. Afterward, the height of the pull head was adjusted until the pull force reading of the pull-off instrument was 0. Pull force was applied to the pull head through the handle until the asphalt breaks. In the breaking process of asphalt, the tensile strength can be recorded by a digital display sensor. The tensile strength of each asphalt sample and limestone was tested in three replicates to obtain an average value.

The results of the pull-off experiment can directly evaluate the adhesion strength of each asphalt sample and limestone. The tensile strength (*σ*) of each asphalt sample and limestone can be calculated by Equation (6).

(6) $$\sigma =\frac{F_{d}}{A}$$

where *F_d_* is the maximum tensile force when the asphalt is broken. *A* is the contact area of asphalt and limestone cube.

#### 2.3.4. Contact Angle Experiment

**Figure 9:**
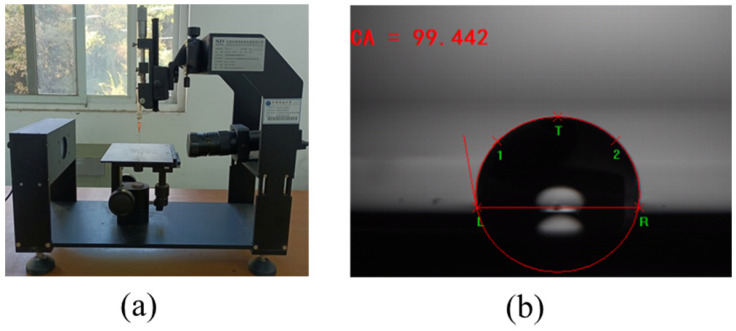
(**a**) The photo of contact angle instrument; (**b**) The photo of a liquid drop on the sample surface.

The SDC-100 contact angle instrument was used in this experiment. The photo of the contact angle instrument and the liquid drop on the sample surface is shown in Figure 9. The sessile drop method was used to measure the contact angles of water and glycerin on the asphalt surface. First, the asphalt sample and glass slide were heated in an oven at 170 °C. When the asphalt became liquid, the asphalt was dropped on to the glass slide. When the asphalt spreads evenly over the entire surface of the glass slide, the glass slide was cooled at room temperature until the asphalt became solid. Water and glycerin with known surface energy parameters were used in the contact angle experiment to calculate the surface free energy of each asphalt sample and limestone. The surface free energy parameters of water and glycerin are listed in Table 5 [30]. The contact angle of water and glycerin on each sample’s surface was measured three times to obtain an average value.

In Table 5, $\gamma_{f}^{d}$ is the dispersion component of the water or glycerol; $\gamma_{f}^{p}$ is polar component of the water or glycerol; *γ* is the surface free energy.

#### 2.3.5. FTIR Experiment

The FTIR of 90# matrix asphalt, WWMA-3, and WWMA-4, were tested in this study. First, each sample was dissolved in carbon disulfide. Then, the prepared solution was taken out and dropped on to the potassium bromide wafer. After waiting for the carbon disulfide to volatilize completely, the FTIR of the samples was measured. The ordinate and abscissa of the FTIR are transmittance and wave number, respectively. The infrared spectrum scanned from 4000 to 500 cm^−1^ wavenumbers. The number of scans was 64 times and the resolution was 4 cm^−1^.

## 3. Results and Discussion

### 3.1. The Rationality Verification of the Asphalt Model

The density and solubility parameters of the optimized asphalt models are listed in Table 6. The actual asphalt density is 1.00–1.04 g/cm^3^ [31]. After geometric optimization and dynamics simulation, their density is in the range of 0.981–0.987 g/cm^3^. Compared with the actual asphalt, the difference value in density between the asphalt models and the actual asphalt is within 5%. The actual reference value of asphalt solubility is 15.3–23.0 (J/cm^3^)^0.5^. The solubility range of the asphalt models constructed is 17.398–17.697 (J/cm^3^)^0.5^ within the reference value range. It shows that the constructed asphalt models can reflect the properties of the natural asphalt from the perspective of density and solubility parameters.

### 3.2. Adhesion Work between Asphalt and Aggregate Oxide

**Figure 10:**
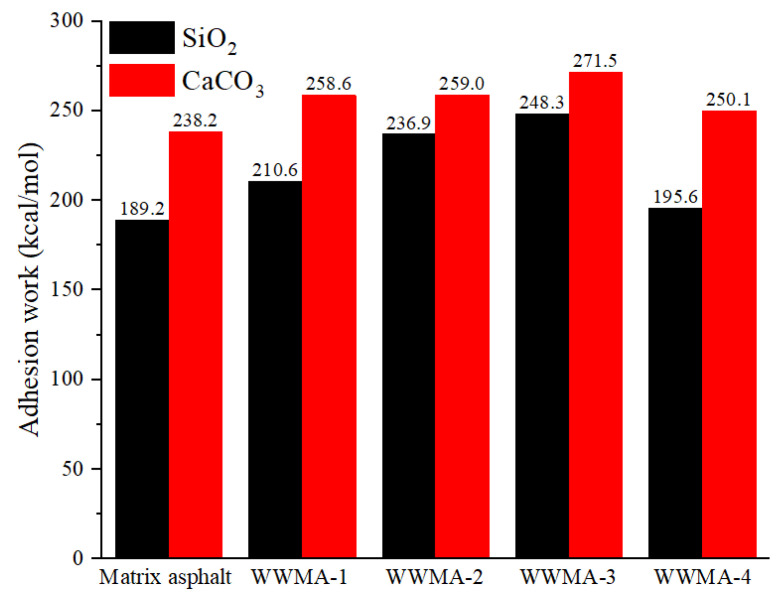
The adhesion work of each asphalt with SiO_2_ and CaCO_3_.

**Figure 11:**
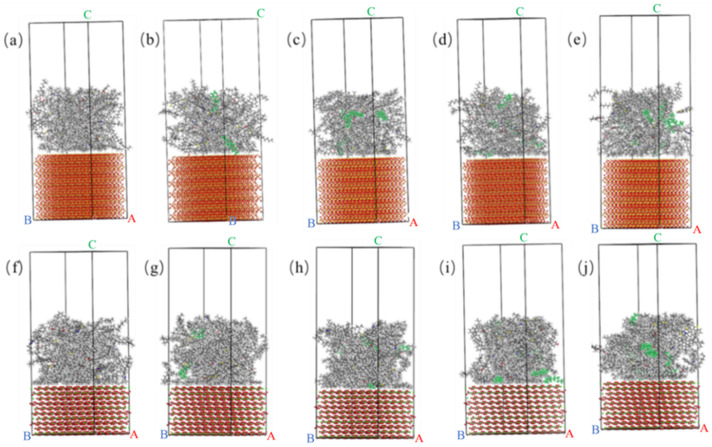
(**a**) Matrix asphalt and SiO_2_ interface model; (**b**) WWMA-1 and SiO_2_ interface model; (**c**) WWMA-2 and SiO_2_ interface model; (**d**) WWMA-3 and SiO_2_ interface model; (**e**) WWMA-4 and SiO_2_ interface model; (**f**) MA and CaCO_3_ interface model; (**g**) WWMA-1 and CaCO_3_ interface model; (**h**) WWMA-2 and CaCO_3_ interface model; (**i**) WWMA-3 and CaCO_3_ interface model; (**j**) WWMA-4 and CaCO_3_ interface model.

The adhesion work of each asphalt and two aggregate oxides is shown in Figure 10. It can be concluded that the WWMA and the two aggregate oxides have all increased compared with the matrix asphalt. With the increase of wax modifier, the adhesion works of asphalt and two aggregate oxides first increase and then decrease. When the content of wax modifier in asphalt increases to 3 wt%, the adhesion work of WWMA with SiO_2_ and CaCO_3_ increased to 248.3 kcal/mol and 271.5 kcal/mol, respectively. Besides, the adhesion works of WWMA-3 with SiO_2_ and CaCO_3_ are higher than those of others. This shows that 3 wt% wax modifier improves the adhesion strength of asphalt with SiO_2_ and CaCO_3_ most significantly. In addition, it can also be seen that the adhesion work between asphalt and CaCO_3_ is greater than that between asphalt and SiO_2_. This indicates that the adhesion strength of asphalt and CaCO_3_ is higher than that of asphalt and SiO_2_. This is consistent with the results of previous laboratory measurements [32,33]. The optimized interface models between each asphalt and two aggregate oxides are shown in Figure 11. It can be seen that the interface distance between asphalt and SiO_2_ is greater than that between asphalt and CaCO_3_. This indicates that the adhesion strength of asphalt and SiO_2_ is less than that of asphalt and CaCO_3_. This is because the adhesion strength between aggregate and asphalt is determined by their atomic interaction force. The atomic interaction force mainly includes Coulomb electrostatic force and van der Waals force [34]. The increase of the interface distance reduces the Coulomb electrostatic force and van der Waals force, resulting in that the adhesion strength of asphalt and CaCO_3_ is better than that of SiO_2_. The follow-up of this article will systematically study the adhesion mechanism of asphalt and CaCO_3_.

### 3.3. Relative Concentration Analysis

**Figure 12:**
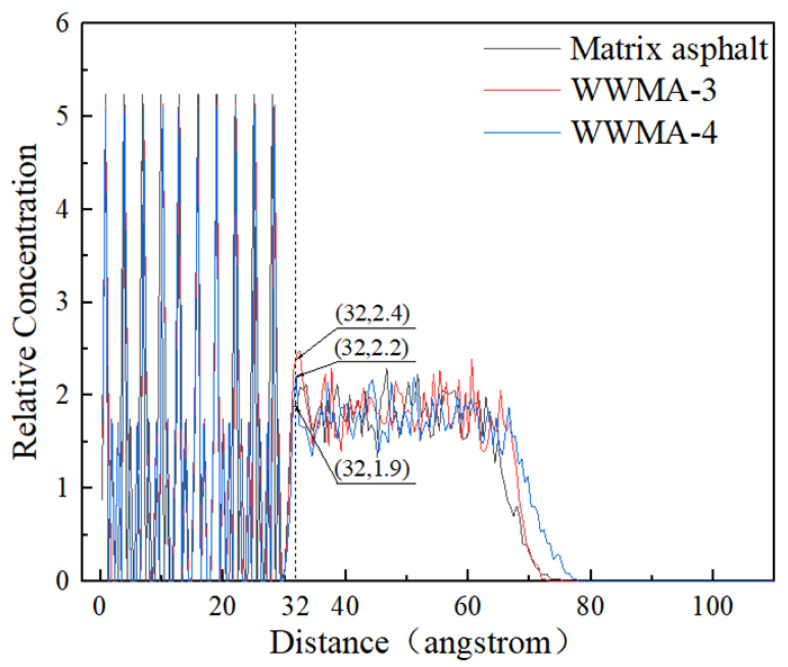
The relative concentration distribution of matrix asphalt, WWMA-3, WWMA-4, and CaCO_3_.

In order to explore the adhesion mechanism of WWMA and aggregate, the relative concentration curves of matrix asphalt, WWMA-3, WWMA-4, and CaCO_3_ in the Z direction, derived in the MS software, are shown in Figure 12. The relative concentration at the distance of 32 angstroms correspond to asphalt molecules. The relative concentration of matrix asphalt, WWMA-3, and WWMA-4, is 1.9, 2.2, and 2.4, respectively. It can be seen that the relative concentration of WWMA-3 is larger than that of matrix asphalt and WWMA-4. This phenomenon indicates that the interaction force between WWMA-3 and aggregate is greater than that of matrix asphalt and WWMA-4. In other words, the interaction force between WWMA-3 and aggregate attracts more asphalt molecules at the asphalt-aggregate interface. This also explains that the adhesion strength between WWMA-3 and CaCO_3_ in Figure 10 is higher than those between other WWMA samples and CaCO_3_.

### 3.4. Pull-Off Test Results

**Figure 13:**
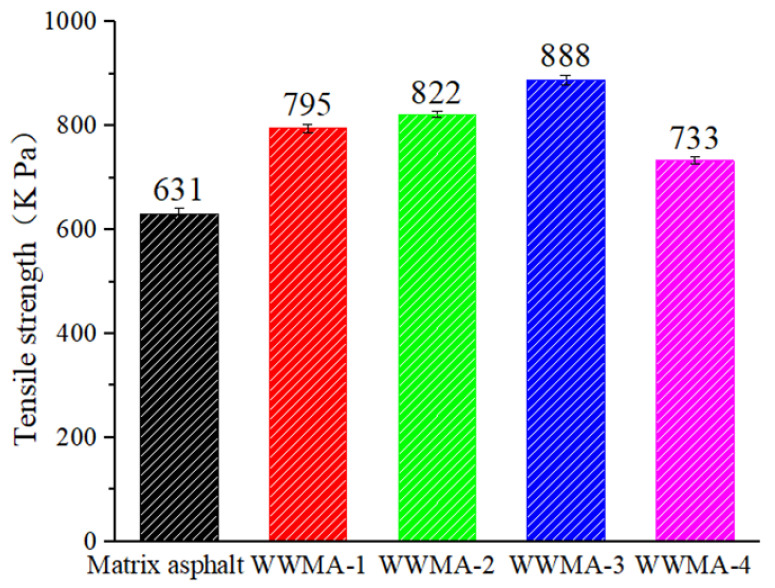
Tensile strength of asphalt and limestone cube.

The tensile strength of asphalt and limestone cube are shown in Figure 13. It can be seen that the tensile strength of the matrix asphalt is 631 kPa. The tensile strength of WWMA and limestone cube is higher than that of matrix asphalt and limestone cube. In particular, the tensile strength of WWMA-3 and limestone cube is increased to 888 kPa, which is 40.7% higher than that of matrix asphalt and limestone cube. In addition, it can be seen that the tensile strength of asphalt and limestone cube first increases and then decreases with the increase of wax modifier. WWMA-3 has a higher tensile strength with limestone cube than other asphalts, indicating that WWMA-3 has the best adhesion strength with limestone cube. Pull-off test results are in good agreement with the MD simulation results. This proves that MD can simulate the adhesion performance between asphalt and aggregate well.

### 3.5. Contact Angle Experiment

The surface free energy theory can be used to study the adhesion of asphalt and aggregate [35,36]. The surface tension of the solid or liquid is composed of the dispersion component caused by the Newtonian force, and the polar component caused by the non-Newtonian force [37,38]. Contact angles of two liquids (water and glycerin) on the surface of all asphalt samples can be measured by a contact angle instrument to calculate the surface free energy of the asphalt. Contact angles of the two liquids on the surface of all asphalt samples are listed in Table 7.

In Table 7, it can be seen that contact angles of water and glycerin on the WWMA surface have become smaller, compared with those on the matrix asphalt surface. The contact angles of water and glycerin on the WWMA-3 surface are 94.31° and 95.79°. These contact angles are lower than those on the other asphalt surfaces. It indicates that the wax modifier improves the hydrophilicity of the asphalt.

According to the measured contact angles of water and glycerin on all asphalt surfaces in Table 7, the surface free energy parameters of asphalt and limestone cube can be calculated by Equations (7) and (8) [39].

(7) $$\frac{\gamma_{f}}{2\sqrt{\gamma_{f}^{d}}}\cdot (1+cos\theta )=\sqrt{\frac{\gamma_{f}^{p}}{\gamma_{f}^{d}}}\cdot \sqrt{\gamma_{a}^{p}}+\sqrt{\gamma_{a}^{d}}$$

(8) $$\gamma =\gamma^{d}+\gamma^{p}$$

In Equation (7): $\gamma_{f}$ is the surface energy of the liquid; $\gamma_{a}^{d}$ is the dispersion component of the solid; $\gamma_{a}^{p}$ is polar component of the solid; $\gamma_{f}^{d}$ is dispersion component of liquid; $\gamma_{f}^{p}$ is polar component of the liquid; *θ* is contact angle of the liquid on the solid surface. In Equation (8): $\gamma^{p}$ is the polar component of the asphalt or limestone, and it has the same meaning as $\gamma_{a}^{p}$; $\gamma^{d}$ is the dispersion component of the asphalt or limestone, and it has the same meaning as $\gamma_{a}^{d}$; *γ* is the indicated surface free energy of the asphalt or limestone.

**Figure 14:**
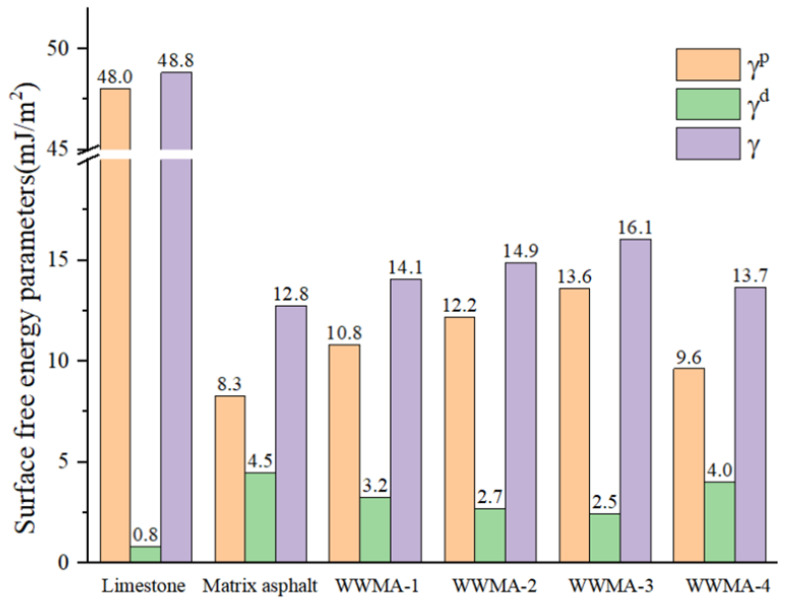
The surface free parameters of asphalt samples and limestone.

The surface free energy parameters of all asphalt samples and limestone are shown in Figure 14. It can be seen that the surface free energy of the WWMA is significantly higher than that of the matrix asphalt. It is worth noting that the surface free energy of the WWMA-3 is 16.1 mJ/m^2^, which is higher than those of other asphalts.

From a microscopic point of view, the adhesion work is expressed as the energy required to separate the asphalt from the aggregate. According to the dispersion and polar components obtained in Figure 14, the adhesion work (*W_adhesion_*) of asphalt and limestone can be calculated by Equation (9) [34]:

(9) $$W_{adhesion}=2\sqrt{\gamma_{b}^{d}\gamma_{lim}^{d}}+\sqrt{\gamma_{b}^{p}\gamma_{lim}^{p}}$$

where $\gamma_{lim}^{p}$ is the polar component of the limestone cube; $\gamma_{lim}^{d}$ is the dispersed component of the limestone cube; $\gamma_{b}^{p}$ is the polar component of the asphalt; $\gamma_{b}^{d}$ is the dispersed component of the asphalt.

**Figure 15:**
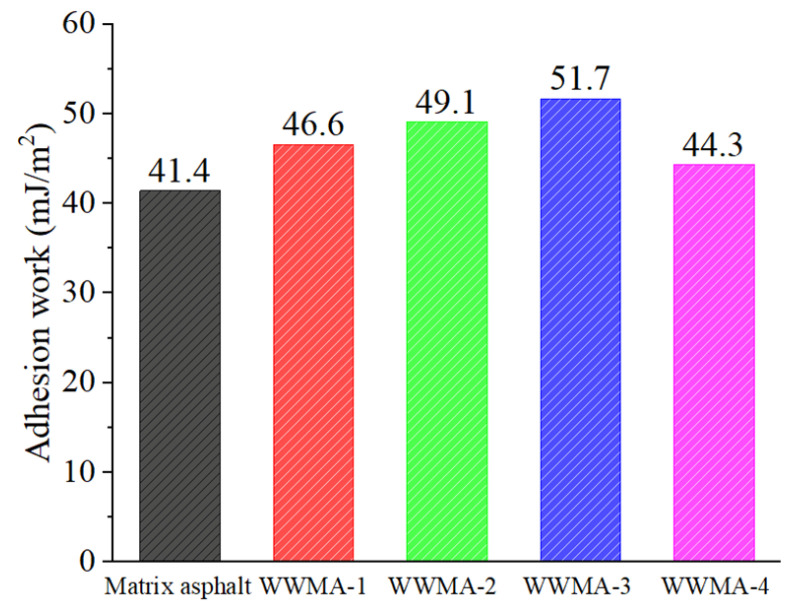
Calculated adhesion work of each asphalt and limestone by the contact angle test.

The calculated adhesion works of asphalts and limestone are shown in Figure 15. It can be seen that the adhesion work of the WWMA and limestone is higher than that of the matrix asphalt and limestone. This is because the wax modifier increases the polar component of the asphalt in Figure 14. In Equation (9), it can be seen that the adhesion work of asphalt and limestone is positively related to the polar components of asphalt. Compared with matrix asphalt, the adhesion work of WWMA-3 and limestone has increased by 10.3 mJ/m^2^. WWMA-3 has a higher adhesion work with limestone than other asphalts. It indicates that WWMA-3 has the best adhesion strength with limestone. The results of the contact angle experiment also verify the accuracy of the MD simulation calculation results.

### 3.6. FTIR

**Figure 16:**
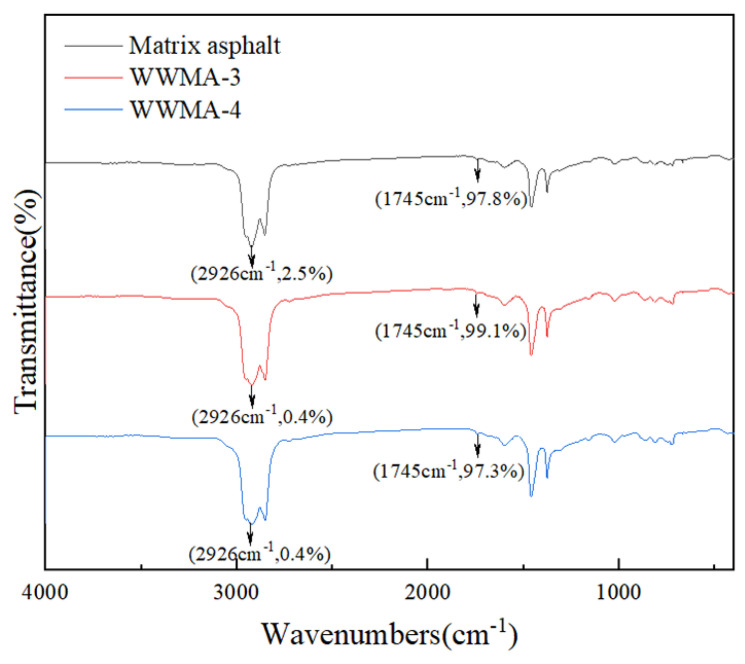
The FTIR spectra of matrix asphalt, WWMA-3 and WWMA-4.

The FTIR spectra of matrix asphalt, WWMA-3, and WWMA-4, are shown in Figure 16. The relative transmittance of the carbonyl group was calculated according to the reference method [40]. The relative transmittance of the carbonyl group can be calculated by Equation (10):

(10) $$R_{C=O}=\frac{Tr_{C=O}}{Tr_{-CH2-}}$$

where $Tr_{C=O}$ is the transmittance of the carbonyl group; $Tr_{-CH2-}$ is the transmittance of the methylene group.

The peak at 1745 cm^−1^ and 2926 cm^−1^ is ascribed to the carbonyl group and methylene [41,42]. The relative transmittances of the carbonyl group of matrix asphalt, WWMA-3, and WWMA-4, are 39.12, 247.75, and 243.25, respectively. It indicates the relative carbonyl content of WWMA-3 and WWMA-4 are both higher than that of matrix asphalt. The electronegativity of oxygen in the carbonyl group is higher than that of carbon, so the carbonyl group is negatively charged. In CaCO_3_, Ca^2+^ is positively charged with +2 valence. This may lead to the mutual attraction of carbonyl and Ca^2+^ to improve the adhesion of asphalt and aggregate. In addition, it can be seen that the relative carbonyl content in WWMA-3 is higher than those in matrix asphalt and WWMA-4. This reveals the inherent mechanism that the adhesion strength of WWMA-3 and CaCO_3_ is higher than that of matrix asphalt and WWMA-4. Based on the above calculated adhesion work, it can be inferred that the adhesion work between asphalt and aggregate is positively correlated with the relative carbonyl content.

## 4. Conclusions

This paper uses MD simulation to study the adhesion of WWMA and aggregate. Two typical aggregate oxides (SiO_2_ and CaCO_3_) were selected to construct the asphalt-aggregate interface model. The molecular interaction between WWMA-aggregate was quantitatively studied with MD simulation. Pull-off and contact angle experiments verify the simulation result. The conclusions are as follows:

(1) In MD simulation, the adhesion work of the WWMA and the two aggregate oxides is larger than that of matrix asphalt and the two aggregate oxides. The adhesion works of the WWMA-3 on SiO_2_ and CaCO_3_ increase to the peak values. They increase by 31.2% and 14.0% compared to the matrix asphalt on SiO_2_ and CaCO_3_. The pull-off experiments and the contact angle experiments are also in good agreement with that of the MD simulation.
(2) The pull-off and contact angle experiments prove that WWMA can increase the tensile strength and adhesion work between the asphalt and the limestone cube. The tensile strength and the adhesion work between the aggregate and the asphalt containing 3 wt% wax modifier reach the peak values. These values are 140.7% and 124.9% compared with those between the aggregate and the matrix asphalt.
(3) FTIR reveals that the carbonyl content in WWMA is much higher than that in matrix asphalt. It explains well that carbonyl can enhance the adhesion strength of asphalt and aggregate.

In this paper, the performances of WWMA are only studied indoors, and field experiments will be a new direction to further prove the accuracy of MD simulation results.

## Figures and Tables

**Table 1:** Matrix asphalt model parameters.

Molecules in Model		Molar Mass	Molecular Formula	Number of Molecules
Group	Label			
Saturate	A1	422.9	C_30_H_62_	4
	B1	483.0	C_35_H_62_	4
Aromatic	A2	464.8	C_35_H_44_	11
	B2	406.8	C_30_H_46_	13
Resin	A3	554.0	C_40_H_59_N	4
	B3	573.1	C_40_H_60_S	4
	C3	414.8	C_29_H_50_O	5
	D3	530.9	C_36_H_57_N	4
	E3	290.4	C_18_H_10_S_2_	15
Asphaltene	AS1	575.0	C_42_H_54_O	3
	AS2	888.5	C_66_H_81_N	2
	AS3	707.2	C_51_H_63_S	3

**Table 2:** Single-cell model parameters of SiO_2_ and CaCO_3_.

Oxide Type	Edge Length (Å)			Cross Angle (°)		
	a	b	c	α	β	γ
SiO_2_	4.98	4.98	6.95	90	90	90
CaCO_3_	4.99	4.99	17.06	90	90	120

**Table 3:** Physical properties of 90# matrix asphalt.

Physical Properties	Unit	Value	Technical Requirements	Test Method (JTG F40-2004)
Softening Point	°C	49.6	>42	T0606
Penetration (25°)	0.1 mm	86.1	80–100	T0604
Ductility (15°)	mm	153	>100	T0605
Flashing point	°C	345	>245	T0611

**Table 4:** The physical properties of wax modifier.

Physical Properties	Unit	Value
Density	g/cm^3^	0.9
Melting point	°C	99
Flash point	°C	285

**Table 5:** Surface free energy parameters of water and glycerin (25 °C, unit: mJ/m^2^).

Surface Free Energy Parameters	Water	Glycerol
$\gamma_{f}^{d}$	21.8	37.0
$\gamma_{f}^{p}$	51.0	26.4
*γ*	72.8	63.4

**Table 6:** Density of matrix asphalt and WWMA.

Sample	Density (g/cm^3^)	Reference Value (g/cm^3^)	Solubility Parameter (J/cm^3^)^0.5^	Reference Value (J/cm^3^)^0.5^
Matrix asphalt	0.987	1.00–1.04	17.398	15.3–23.0
WWMA-1	0.986		17.586	
WWMA-2	0.983		17.544	
WWMA-3	0.981		17.514	
WWMA-4	0.983		17.697	

**Table 7:** Contact angles of the liquid on the surface of all asphalt samples (°).

Sample	limestone	Matrix Asphalt	WWMA-1	WWMA-2	WWMA-3	WWMA-4
Water	61.71	99.44	97.09	96.01	94.31	97.67
Glycerin	73.02	97.33	96.95	96.85	95.79	96.39

## Data Availability

The data presented in this study are available on request from the corresponding author.
